# Smoking cessation mutually facilitates alcohol drinking cessation among tobacco and alcohol co-users: A cross-sectional study in a rural area of Shanghai, China

**DOI:** 10.18332/tid/114076

**Published:** 2019-11-25

**Authors:** Ruiping Wang, Bin Li, Yonggen Jiang, Ying Guan, Guimin Wang, Genming Zhao

**Affiliations:** 1 Yueyang Hospital, Shanghai University of Traditional Chinese Medicine, Shanghai, China; 2 Disease Control Division, Songjiang Center for Disease Control and Prevention, Shanghai, China; 3 School of Public Health, Fudan University, Shanghai, China

**Keywords:** smoking cessation, drinking cessation, tobacco and alcohol co-user, mutually, facilitate

## Abstract

**INTRODUCTION:**

Tobacco smoking and alcohol drinking are strongly paired behaviours, affecting millions of people worldwide. Studies in western countries demonstrate that alcohol use among smokers makes it harder to quit smoking, and addressing alcohol use is particularly important for smoking cessation, but the evidence is limited in China. We conducted a cross-sectional study to understand the prevalence of smoking, drinking, as well as tobacco and alcohol co-use, and to explore how smoking cessation mutually facilitates drinking cessation among tobacco and alcohol co-users.

**METHODS:**

During 2016 and 2017, we sampled 36698 participants aged >18 years in Songjiang district, Shanghai. A questionnaire was designed to collect data, and participants were classified into non-smokers and smokers (current and former smokers), as well as non-alcohol drinkers and alcohol drinkers (current and former alcohol drinkers). SAS software was applied to analyse the differences by weighted logistic regressions.

**RESULTS:**

The prevalence of tobacco smoking, alcohol drinking, and tobacco and alcohol co-use was 23.53%, 13.52% and 9.85%, respectively. Smoking cessation prevalence was 15.93%, which was higher than drinking cessation prevalence (8.22%). Tobacco and alcohol co-users had a higher prevalence of smoking cessation (16.95%) than participants who were only smokers (15.20%) and had higher prevalence of alcohol drinking cessation (8.71%) than residents who were only drinkers (6.91%). Tobacco and alcohol co-users who stopped alcohol drinking were much more likely to stop smoking than those who still drank alcohol (OR=8.83; 95% CI: 6.91–11.28) or those who only smoked (OR=7.51; 95% CI: 5.93–9.52).

**CONCLUSIONS:**

Drinking cessation prevalence was lower than that of smoking cessation, and drinking cessation could mutually facilitate smoking cessation among tobacco and alcohol co-users. Tobacco smoking cessation programs could incorporate alcohol drinking cessation measures to achieve higher public health benefits.

## INTRODUCTION

Studies have indicated that tobacco smoking and alcohol drinking are strongly paired behaviours, and smoking and drinking affect millions of people worldwide^1,2^. The harmful role of tobacco in pulmonary, cardiovascular and cancer diseases has been well established^3,4^, but currently many people believe that regular consumption of a small quantity of alcohol is beneficial to health and do not perceive alcohol drinking as a harmful habit^5,6^. A recently published study in Lancet that analyzed 599912 current drinkers in 83 prospective studies indicated that a threshold for lower risk of all-cause mortality did not exist^7^, and the combined negative effects of drinking and smoking on health outcomes were substantial. It is likely that the combination of nicotine and alcohol has a synergistic effect^8^, and a multiplicative effect operates when smoking is combined with drinking, especially heavy drinking. In comparison with the smoking only, drinking, or neither smoking nor drinking group, the combined effect of nicotine and alcohol poses a markedly greater risk for oral, pharyngeal, laryngeal and oesophagal cancers, as well as for brain morphology and function^9,10^.

China has a rich culture and long history in substance consumption, including alcohol drinking and tobacco smoking^11^. Alcohol is commonly viewed as a symbol of festivity and celebration, and the prevalence of alcohol consumption is around 34% in China^12^. However, health issues related to alcohol consumption such as accidents, violence, and chronic diseases especially, have been largely neglected by the Chinese government^13^. Besides alcohol, the other commonly used substance in China is tobacco^14^. The Global Adult Tobacco Survey (GATS) conducted in 2010 has indicated that 300 million adults in China are current smokers, and around 1 million deaths are attributed to tobacco consumption each year^15-17^. The high prevalence of tobacco and alcohol consumption leads to a heavy disease burden in China.

Many studies in western countries have demonstrated that greater alcohol use among smokers makes it harder for them to quit smoking, and addressing alcohol use is particularly important for smoking cessation^18-20^. Dawson et al.^19^ reported that those who stopped drinking were less likely to be current smokers, and the odds of quitting drinking were roughly tripled among individuals who stopped smoking during the follow-up interval. Other studies also found an inverse association of drinking cessation with current smoking^20-22^. However, to our knowledge, the relationship between smoking cessation and drinking cessation among co-users of alcohol and tobacco is unclear in China.

We conducted a cross-sectional study in Shanghai, China. Our aim was: 1) to investigate the prevalence of tobacco smoking, alcohol drinking, and co-use of tobacco and alcohol, and 2) to explore whether smoking cessation and alcohol cessation mutually facilitate each other among co-users of tobacco and alcohol.

## METHODS

### Study population

We conducted a cross-sectional study in Songjiang District during 2016 and 2017. We employed multistage sampling to recruit the study population in Songjiang. In stage one, we randomly selected 4 out of the 15 sub-districts in Songjiang District: ZS, XQ, SS and MG. In stage two, there were 9, 18, 4 and 16 neighbourhood communities that were randomly selected from ZS, XQ, SS and MG subdistrict, respectively (nearly 60% of neighbourhood communities in each sub-district). In total, 47 neighbourhood communities were selected. In stage three, we recruited individuals aged ≥18 years and that had lived in Songjiang district for more than five years in each of the selected neighbourhood communities. Overall, 37543 residents were sampled and invited to participate in this study. The ethics approval was authorized and approved by Fudan University Institution Review Board (No.IRB#2016-04-0586), and an informed consent form was signed by each participant before the questionnaire interview. Information that could identify individual participants during or after data collection could not be accessed by the authors. Finally, 36698 residents completed the interview and were included in the analyses.

### Data collection

The questionnaire for data collection was designed by the School of Public Health, Fudan University. A pilot study demonstrated that the split-half reliability coefficient of the questionnaire was 0.79 and the content validity coefficient was 0.87. Interviews were conducted face-to-face with each participant using a structured questionnaire. Interviewers used self-designed Android pad-assisted software to collect information. We also audio recorded the interview process for subsequent inspection. Ten quality controllers were equipped at each selected sub-district, who were responsible for examining the content quality of uploaded questionnaires and uploaded audio-recording files.

The questionnaire included four parts. Part A covered eight questions of demographic information. Part B covered 10 tobacco-related chronic diseases information. Part C covered 36 health-related behaviour items, including tobacco use, alcohol consumption, and sleep habit. Among them, we designed 14 questions to collect tobacco consumption information, for example: ‘Have you ever smoked at least one cigarette every day for over six months?’, ‘What was your age when you smoked the first cigarette?’, ‘Do you still smoke now?’, ‘What was your age when you quit smoking?’, ‘How many cigarettes do you smoke each day?’ etc. and six questions to collect alcohol drinking information, for example: ‘Have you ever drank at least three times a week for over six months?’, ‘What was your age when you began alcohol drinking?’, ‘Do you still drink alcohol now?’, ‘What was your age when you quit drinking?’, ‘How many grams of alcohol do you drink on average each week?’ etc. Part D covered personal contact information both for the investigator and the participant.

### Definition and index calculation

We defined smokers as people who smoked at least one cigarette per day for six months or more, in their lifetime^11,15,23^. Current smokers were defined as people who still smoked at the time of investigation; former smokers were defined as people who had quit smoking for over three months at the time of investigation; heavy smokers were defined as smokers who smoked 20 cigarettes or more per day. The prevalence of smoking was calculated as the number of smokers divided by the total number of participants, and the prevalence of smoking cessation was calculated as the number of former smokers divided by the number of smokers. Likewise, drinkers were defined as people who drank alcohol at least three times a week for six months or more in their lifetime. Current drinkers were defined as people who still drank at the time of investigation; former drinkers were defined as people who had quit drinking for over 3 months at the time of investigation; heavy drinkers were defined as drinkers who drank over 2000 g alcohol each week. The prevalence of drinking was calculated as the number of drinkers divided by the total number of participants, and the prevalence of drinking cessation was calculated as the number of former drinkers divided by the number of drinkers. Tobacco and alcohol co-users were defined as people who had both smoking behavior and drinking behavior. We divided smokers and drinkers into three types: 1) participants who were tobacco smokers and alcohol drinkers (Type A); participants who were tobacco smokers but not alcohol drinkers (Type B); and participants who were alcohol drinkers but not tobacco smokers (Type C). To explore the relationship between smoking cessation and drinking cessation, we divided participants of Type A into two groups: 315 participants with drinking cessation (DC) and 3301 participants without DC. We compared the smoking cessation prevalence between participants of Type A with DC and without DC, and between participants of Type A with DC and participants of Type B. We also divided participants of Type A into 613 participants with smoking cessation (SC) and 3003 participants without SC, then we compared the drinking cessation prevalence between participants of Type A with SC and without SC, and between participants of Type A with SC and participants of Type C. Age was divided into six age groups (18–29, 30–39, 40–49, 50–59, 60–69 and 70–92 years). Education was recorded as completed years of schooling and categorized into five categories: 0 years (illiterate), 1–6 years (primary school), 7–9 years (junior high school), 10–12 years (senior high school), and >12 years (college and above).

### Data analysis

Data analysis was performed by SAS software (version 9.2). We described the data using frequency counts and proportions (prevalence) for qualitative variables, and means and standard deviation (SD) for quantitative variables. We applied weighted logistic regression to calculate the odds ratios (OR) and 95% confidence interval (95% CI) for smoking behaviour, drinking behaviour, smoking cessation, and drinking cessation among people in the three types, among residents with different demographic characteristics. We applied chi-squared tests to examine differences of demographic characteristics among residents of Type A, Type B and Type C. Weighted logistic regression was applied to calculate OR and 95% CI of smoking cessation prevalence between residents of Type A with and without DC, as well as between residents of Type A with DC and residents of Type B. We also applied weighted logistic regression to calculate the OR and 95% CI of drinking cessation prevalence between residents of Type A with and without SC, as well as between residents of Type A with SC and residents of Type C. Figures were produced to compare the prevalence of smoking cessation and drinking cessation among residents of Type A, Type B and Type C. A p-value <0.05 (two-tailed) was considered as statistically significant.

## RESULTS

Of the 36698 participants included in this analysis, 40.63% (14912) were male. The age ranged 18–92 years, with an average age of (56.37±11.29) years. The majority of residents were married (92.83%), and over 80% of residents had an education under junior high school (<12 years).

### Prevalence of tobacco smoking and alcohol drinking

Among 36698 participants, 8636 (23.53%) were ever smokers and 5501 of these were heavy smokers. The prevalence of smoking was significantly higher in males (57.48%) than in females (0.30%). Participants aged >40 years had a higher prevalence of smoking than participants <40 years. Participants with primary and high school (junior and senior) education had higher smoking prevalence than participants who were illiterate or had better than college education. Married as well as widows/widowers had higher prevalence of smoking than those who were unmarried or divorced (Table 1).

**Table 1:** The prevalence of tobacco smoking and alcohol drinking among residents with different demographic characteristics in a rural area of Shanghai, China

*Variables*	*Tobacco smoker*						*Alcohol drinker*					
	
	*Yes n (%)*	*No n (%)*	*OR*	*95% CI*	OR*	95% CI*	*Yes n (%)*	*No n (%)*	*OR*	*95% CI*	OR*	95% CI*
**Gender**												
Female	65 (0.30)	21721 (99.70)	1.00	-	-	-	166 (0.76)	21620 (99.24)	1.00	-	-	-
Male	8571 (57.48)	6341 (42.52)	451.66	353.30-577.40	-	-	4796 (32.16)	10116 (67.84)	61.75	52.81-72.21	-	-
**Age** (years)												
18–29	118 (11.49)	909 (88.51)	1.00	-	1.00	-	33 (3.21)	994 (96.79)	1.00	-	1.00	-
30–39	427 (16.58)	2149 (83.42)	1.53	1.23-1.90	2.07	1.62-2.66	194 (7.53)	2382 (92.47)	2.45	1.68-3.57	2.95	2.00-4.35
40–49	1005 (21.85)	3595 (78.15)	2.15	1.76-2.64	3.64	2.88-4.60	502 (10.91)	4098 (89.09)	3.69	2.58-5.28	4.77	3.29-6.90
50–59	2777 (23.11)	9238 (76.89)	2.32	1.91-2.82	4.31	3.45-5.38	1513 (12.59)	10502 (87.41)	4.34	3.06-6.16	5.93	4.13-8.49
60–69	3418 (26.88)	9296 (73.12)	2.83	2.33-3.45	3.65	2.93-4.55	2126 (16.72)	10588 (83.28)	6.05	4.26-8.58	6.65	4.65-9.52
70–92	891 (23.66)	2875 (76.34)	2.39	1.94-2.93	2.58	2.02-3.22	594 (15.77)	3172 (84.23)	5.64	3.94-8.07	5.79	4.00-8.36
**Education**												
Illiterate	546 (10.13)	4846 (89.87)	1.00	-	1.00	-	384 (7.12)	5008 (92.88)	1.00	-	1.00	-
Primary	2951 (25.05)	8830 (74.95)	2.97	2.69-3.27	1.17	1.02-1.34	1882 (15.97)	9899 (84.03)	2.48	2.21-2.78	1.04	0.91-1.19
Junior High	3611 (27.83)	9362 (72.17)	3.42	3.11-3.77	1.78	1.03-1.35	1961 (15.12)	11012 (84.88)	2.32	2.07-2.61	0.82	0.72-0.94
Senior High	1213 (28.22)	3085 (71.78)	3.49	3.12-3.90	1.03	0.88-1.20	594 (13.82)	3704 (86.18)	2.09	1.83-2.39	0.66	0.57-0.77
College and above	315 (13.98)	1939 (86.02)	1.44	1.24-1.67	0.41	0.34-0.49	141 (6.26)	2113 (93.74)	0.87	0.71-1.06	0.31	0.25-0.38
**Marriage**												
Married	8233 (24.17)	25835 (75.83)	1.00	-	1.00	-	4733 (13.89)	29335 (86.11)	1.00	-	1.00	-
Widow/widower	118 (24.95)	355 (75.05)	1.04	0.85-1.29	2.39	1.66-3.44	56 (11.84)	417 (88.16)	0.83	0.63-1.10	1.13	0.82-1.57
Unmarried	202 (12.23)	1450 (87.77)	0.44	0.38-0.51	0.95	0.77-1.17	135 (8.17)	1517 (91.83)	0.55	0.46-0.66	1.14	0.93-1.40
Divorced	83 (16.44)	422 (83.56)	0.62	0.49-0.78	0.29	0.23-0.38	38 (7.52)	467 (92.48)	0.51	0.36-0.70	0.31	0.22-0.44

* Odds ratio (OR) between group on tobacco smoking and alcohol drinking prevalence after the adjustment of gender.

Alcohol drinking was noted for 4962 out of 36698 participants, and among them 3353 were heavy alcohol drinkers (67.57%). Alcohol drinking prevalence was 13.52%. The prevalence of alcohol drinking was significantly higher in males (32.16%) than in females (0.76%). Those aged >60 years had a higher prevalence of drinking than those <60 years, and those aged 18–29 years had the lowest prevalence of drinking (3.21%). Participants with primary and high school (junior and senior) education had higher prevalence of drinking than those who were illiterate or had better than college education. Married as well as widow/widower participants had higher prevalence of drinking than unmarried or divorced participants. (Table 1).

There were 3616 Type A participants who had both smoking and drinking behaviors, 5020 Type B participants and 1346 Type C participants. The differences of demographic characteristics were all statistically significant between participants of Type A and Type B, Type B and Type C, as well as Type A and Type C, presented in Table 2.

**Table 2:** The demographic characteristics among residents of Type A (who are smokers and drinkers), Type B (who are smokers but not drinkers) and Type C (who are drinkers but not smokers) in a rural area of Shanghai, China

*Variables*	*Tobacco and alcohol co-user (Type A)*		*Tobacco smoker (Type B)*		*Alcohol drinker (Type C)*				
	
	*n*	*%*	*n*	*%*	*n*	*%*	*P_ab_*	*P_ac_*	*P_bc_*
**Gender**							0.001	0.001	0.001
Male	3608	99.78	4963	98.86	1188	88.26			
Female	8	0.22	57	1.14	158	11.74			
**Age** (years)							0.001	0.001	0.001
18–29	25	0.69	93	1.85	8	0.59			
30–39	127	3.51	300	5.98	67	4.98			
40–49	366	10.12	639	12.73	136	10.10			
50–59	1143	31.61	1634	32.55	370	27.49			
60–69	1560	43.14	1858	37.01	566	42.05			
70–92	395	10.92	496	9.88	199	14.78			
**Education**							0.001	0.02	0.001
Illiterate	263	7.27	283	5.64	121	8.99			
Primary	1382	38.22	1569	31.25	500	37.15			
Junior High	1448	40.04	2163	43.09	513	38.11			
Senior High	434	12.00	779	15.52	160	11.89			
College and above	89	2.46	226	4.50	52	3.86			
**Marriage**							0.11	0.03	0.01
Married	3457	95.60	4776	95.14	1276	94.80			
Widow/widower	47	1.30	71	1.41	9	0.67			
Unmarried	88	2.43	114	2.27	47	3.49			
Divorced	24	0.66	59	1.18	14	1.04			

P_ab_: The difference of demographic characteristic between residents of Type A and Type B. P_ac_: The difference of demographic characteristic between residents of Type A and Type C. P_bc_: The difference of demographic characteristic between residents of Type B and Type C.

### Smoking cessation and drinking cessation condition

Smoking cessation prevalence was 15.93% (1376/8636), which was higher than the drinking cessation prevalence (8.22%, 408/4962). Smoking cessation prevalence was higher in participants of Type A (16.95%, 613/3616) than participants of Type B (15.20%, 763/5020) (χ^2^=4.82, p<0.05). The drinking cessation prevalence was significantly higher in participants of Type A (8.71%, 315/3616) than participants of Type C (6.91%, 93/1346) (χ^2^=4.22, p<0.05).

**Figure 1:**
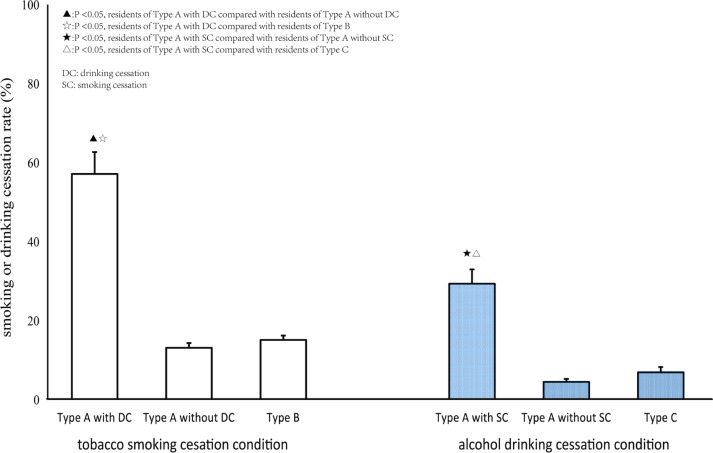
The difference of smoking cessation prevalence between smokers and alcohol & tobacco co-users, as well as the difference of drinking cessation prevalence between drinkers and alcohol & tobacco co-users in Songjiang district, Shanghai, China.

Figure 1 indicates that smoking cessation prevalence was significantly higher in participants of Type A with DC (57.14%) than participants of Type A without DC (13.12%), and higher than participants of Type B as well (15.20%). The drinking cessation prevalence was significantly higher in participants of Type A with SC (29.36%) than participants of Type A without SC (4.5%), and higher than participants of Type C as well (6.91%).

Table 3 demonstrates that heavy alcohol drinkers (13.41%) of Type A had lower smoking cessation prevalence than non-heavy drinkers (25.11%), and heavy tobacco smokers (7.49%) of Type A had lower drinking cessation prevalence than non-heavy smokers (11.31%).

**Table 3:** The prevalence of smoking cessation and drinking cessation among residents of Type A, Type B and Type C with consideration of the amounts of tobacco smoking and alcohol drinking in a rural area of Shanghai, China

*Participant type*	*Smoking cessation prevalence*				*Drinking cessation prevalence*			
	
	*n*	*%*	*χ2*	*p*	*n*	*%*	*χ2*	*p*
**Type A (smoker and drinker)**			73.31	<0.01			29.91	<0.01
Heavy drinker (n=2521)	338	13.41			177	7.02		
Not heavy drinker (n=1095)	275	25.11			138	12.60		
**Type A (smoker and drinker)**			8.50	<0.01			14.49	<0.01
Heavy smoker (n=2458)	386	15.70			184	7.49		
Not heavy smoker (n=1158)	227	19.60			131	11.31		
**Type B (smoker but not drinker)**			59.06	<0.01				
Heavy smoker (n=3043)	367	12.06			-	-		
Not heavy smoker (n=1977)	396	20.03			-	-		
**Type C (drinker but not smoker)**							14.96	<0.01
Heavy drinker (n=832)	-	-			40	4.81		
Not heavy drinker (n=514)	-	-			53	10.31		

### Relationship between smoking cessation and drinking cessation

**Figure 2:**
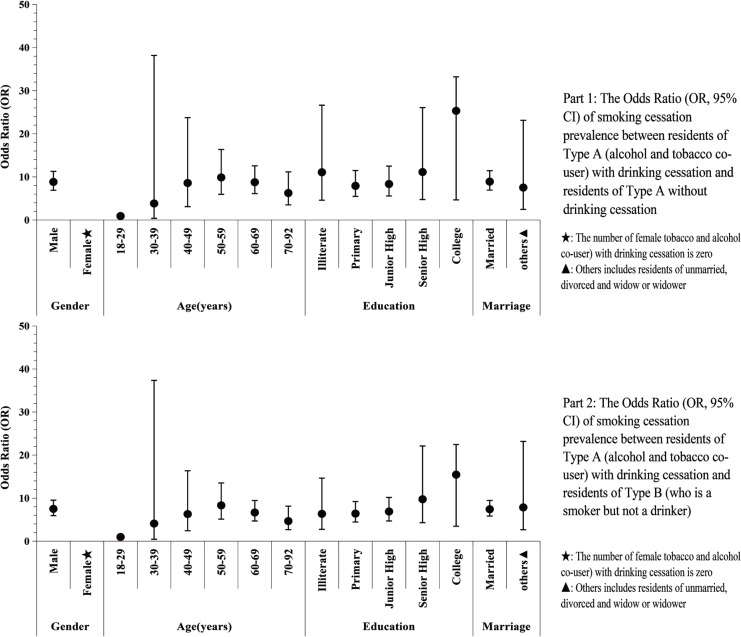
The Odds Ratio (OR, 95% CI) of Smoking cessation prevalence between participants of Type A with drinking cessation and participants of Type A without drinking cessation, and between participants of Type A with drinking cessation and participants of Type B.

Respondents of Type A with DC were more likely to stop smoking than respondents of Type A without DC (OR=8.83; 95% CI: 6.91–11.28), and participants of Type B (OR=7.44; 95% CI: 5.87–9.42). Figure 2 indicates that, except for female participants and participants aged 18–29 years or 30–39 years, residents of Type A with DC were more likely to stop smoking than residents of Type A without DC and residents of Type B, despite their age, education level and marital status (Figure 2).

**Figure 3:**
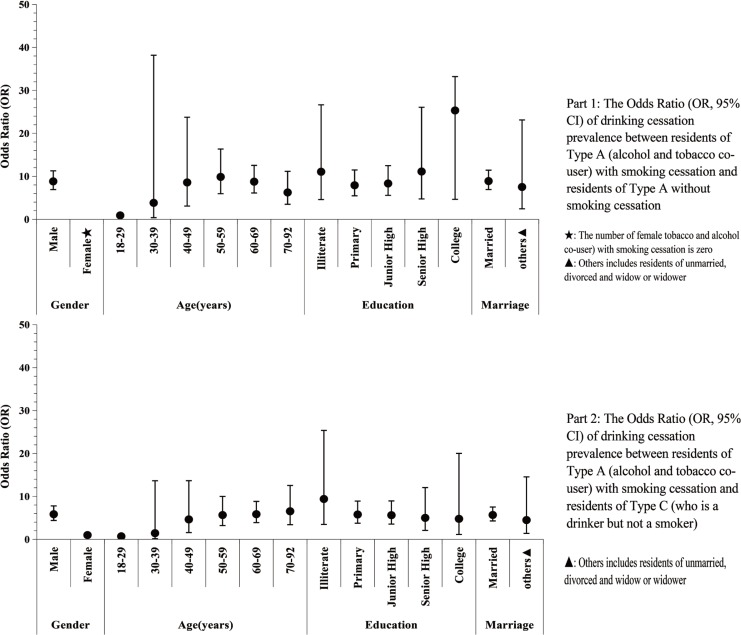
The Odds Ratio (OR, 95% CI) of drinking cessation prevalence between participants of Type A with smoking cessation and participants of Type A without smoking cessation, and between participants of Type A with smoking cessation and participants of Type C.

Tobacco smoking cessation could also facilitate drinking cessation, participants of Type A with SC were more likely to stop drinking than participants of Type A without SC (OR=8.83; 95% CI: 6.91– 11.28), and participants of Type C (OR=5.60; 95% CI: 4.26–7.36). Figure 3 indicates that, except for female participants, participants aged 18–29 years or 30–39 years, participants of Type A with SC were more likely to stop drinking than participants of Type A without SC and participants of Type C, despite their age, education level and marital status (Figure 3).

## DISCUSSION

In this study, smoking cessation prevalence was higher than drinking cessation prevalence. Heavy alcohol drinkers had lower smoking cessation prevalence than non-heavy drinkers, and heavy tobacco smokers had lower drinking cessation prevalence than non-heavy smokers. Smoking cessation and drinking cessation could mutually facilitate each other among tobacco and alcohol co-users, independently of age, marital status and education level.

Tobacco consumption-induced diseases burden is a significant public health issue globally. In this study, the prevalence of smoking in the rural area of Shanghai was 23.53%, which was lower than the migrant monitoring survey in China^16^ in 2013 and the GATS^23^ in 2012. The lower prevalence of smoking might be attributable to the implementation of public health education and intervention on smoking cessation in Shanghai^17^. Meanwhile, many more participants have become aware of the physical harms of tobacco use in recent years and stopped tobacco smoking successfully^24^. However, male participants with a high school education and aged 40–69 years had higher smoking prevalence. These people could be our target population to control smoking behaviour.

Alcohol consumption has a complex association with health and is the leading risk factor for premature death and disability^25-27^. The prevalence of alcohol drinking was 13.52% in this study, consistent with findings in the survey of China Kadoorie Biobank (CKB)^24^, and the Wuhan study in 2015^25^. Despite the fact that the prevalence of drinking in China is lower than in many Western and African populations^2,26,27^, the potential public health challenge induced by alcohol drinking should not be ignored by the Chinese Central Government (CCG). In China, alcohol drinking is traditionally used in festivals, social gatherings and special activities^11,12^. Compared with tobacco use, many people do not perceive alcohol as harmful, and mistakenly believe that the regular consumption of alcohol in small quantities each time could protect against cardiovascular diseases^2,23^.

Meanwhile, there is no law or regulation on alcohol drinking in China, and advertisements of alcohol products are extremely common in magazines, newspapers, films and TV programs. Thus, it is very common in China for people to be unaware of the relationship between alcohol consumption and disease burden induced by alcohol. Growing evidence demonstrates that drinking alcohol leads to increased burden of chronic diseases such as cancers and chronic liver disease including accidents and injuries^11-14,25,26^. The proportion of participants engaging in harmful drinking behaviour has increased in China over the past decades, especially among young men. The Chinese Central Government should enact laws or regulations to control alcohol drinking, especially among young adults.

Some studies demonstrate a lower smoking cessation prevalence in China with over 80% of smokers having no intention to quit smoking^23^. In this study, only 15.93% of smokers quit smoking successfully, consistent with previous studies^28,29^. The lower smoking cessation prevalence could be a combined effect of serious nicotine dependency and better tobacco affordability due to low tobacco prices in China, which hinders smokers from quitting smoking^30^. Moreover, the drinking cessation prevalence is 8.22%, which is lower than the smoking cessation prevalence in the same population. As previously mentioned, alcohol drinking is a widely accepted cultural tradition in China, and there is no regulation or law on alcohol drinking, even for young adults. Moreover, many people perceive alcohol use as harmless and only consider quitting drinking when they encounter health problems^2-4,25^. The ‘sicker-quitter effect’^31^ has been a major reason for alcohol drinking cessation in the Chinese population, which could partially explain the lower drinking cessation rate in this study. We suggest that health-related institutions should provide intense, repeated education about the benefits of drinking and smoking cessation among alcohol and tobacco consumers.

Previous studies demonstrate that smoking and drinking are strongly paired behaviours^1,27,32^, and alcohol use makes it harder to quit smoking^32^. Dawson et al.^19^ reported that smoking cessation was strongly and consistently associated with drinking cessation; the odds of drinking cessation were roughly tripled among individuals who stopped smoking. We noticed that drinking cessation and smoking cessation could mutually facilitate each other among tobacco and alcohol co-users, independent of age, marital status and education level. Tobacco and alcohol co-users who stopped drinking are much more likely to stop smoking than those who still drink alcohol, as well as participants who are only smokers. Likewise, tobacco and alcohol co-users who stopped smoking are much more likely to stop drinking than those who still smoke, and those participants who are only drinkers. Similar findings have been reported elsewhere and are consistent with the association of smoking cessation and drinking cessation in numerous studies^21-23,33^. Both nicotine and alcohol weaken the human immune system^34^, so it is more likely that the combination of nicotine and alcohol exposure has a synergistic effect that makes the co-users more vulnerable to sickness^34,35^. The ‘sicker-quitter effect’^31^ and health concern, prompting the seeking of medical advice^30^ , could promote co-users to cease the use of both substances^21,22^. Alternatively, smoking or drinking cessation could be viewed as a marker of the ability to give up psychoactive and potentially addictive substances, which indicates the ability to stop drinking or smoking successfully. Health Examinees (HEXA) study in Korea^36^, which covered 85323 subjects aged 40–69 years, indicated that ex-smokers had higher prevalence of drinking cessation than current smokers, and ex-drinkers exhibited higher odds of being smoking quitters than current drinkers. It is necessary to combine a tobacco cessation program with some drinking cessation measures, which might achieve higher public health benefit among tobacco and alcohol co-users.

### Limitations and strengths

Our study has some limitations. First, the cross-sectional study design only allows the calculation of prevalence and may induce some information bias. Second, reasons for smoking and drinking cessation were not collected among tobacco smokers and alcohol drinkers, which might reduce the specificity of tobacco and alcohol control measures. Third, based on the observational data, detailed information about the interaction between drinking cessation and smoking cessation was limited. The incorporation of some improvements should be considered in follow-up studies. A key strength of this study is the large sampling population size. We sampled 36698 participants accounting for about 6% of the total local population in Songjiang district, so the study results could be generalised to all residents in Songjiang district or even to other rural areas of Shanghai. Also, demographic, tobacco and alcohol consumption information in this study was collected through a faceto-face interview by an electronic questionnaire, which ensures better data quality.

## CONCLUSIONS

This study is the first attempt to estimate the prevalence of tobacco and alcohol co-use and to explore the relationship of tobacco cessation and drinking cessation in China. Drinking cessation prevalence was found to be lower than that of smoking cessation, and drinking cessation could mutually facilitate smoking cessation among tobacco and alcohol co-users. Tobacco smoking cessation programs could incorporate alcohol drinking cessation measures to achieve higher public health benefits.
